# Influenza‐associated mortality determined from all‐cause mortality, Denmark 2010/11‐2016/17: The FluMOMO model

**DOI:** 10.1111/irv.12564

**Published:** 2018-05-06

**Authors:** Jens Nielsen, Tyra Grove Krause, Kåre Mølbak

**Affiliations:** ^1^ Department of Infectious Disease Epidemiology and Prevention Statens Serum Institut Copenhagen S Denmark; ^2^ Department of Veterinary and Animal Science Faculty of Health and Medical Science, University of Copenhagen Copenhagen Denmark

**Keywords:** EuroMOMO, FluMOMO, influenza, mortality, seasonality, temperature

## Abstract

**Background:**

In temperate zones, all‐cause mortality exhibits a marked seasonality, and influenza represents a major cause of winter excess mortality. We present a statistical model, FluMOMO, which estimate influenza‐associated mortality from all‐cause mortality data and apply it to Danish data from 2010/11 to 2016/17.

**Methods:**

We applied a multivariable time series model with all‐cause mortality as outcome, influenza activity and extreme temperatures as explanatory variables while adjusting for time trend and seasonality. Three indicators of weekly influenza activity (IA) were explored: percentage of consultations for influenza‐like illness (ILI) at primary health care, national percentage of influenza‐positive samples, and the product of ILI percentage and percentage of influenza‐positive specimens in a given week, that is, the Goldstein index.

**Results:**

Independent of the choice of parameter to represent influenza activity, the estimated influenza‐associated mortality showed similar patterns with the Goldstein index being the most conservative. Over the 7 winter seasons, the median influenza‐associated mortality per 100 000 population was 17.6 (range: 0.0‐36.8), 14.1 (0.3‐31.6) and 8.3 (0.0‐25.0) for the 3 indicators, respectively, for all ages.

**Conclusion:**

The FluMOMO model fitted the Danish data well and has the potential to estimate all‐cause influenza‐associated mortality in near real time and could be used as a standardised method in other countries. We recommend using the Goldstein index as the influenza activity indicator in the FluMOMO model. Further work is needed to improve the interpretation of the estimated effects.

## 1 BACKGROUND

Mortality is a basic indicator of health, and monitoring of mortality is fundamental for health planning, risk assessment and public health action. There is a demand for standardised robust and comparable estimates to identify changes in mortality patterns in a timely or near real‐time manner to assess and react on changes.

In temperate zones, all‐cause mortality exhibits a marked seasonality with the highest number of deaths in the winter. Many factors may contribute to seasonality, including acute respiratory tract infections and death from cardio‐vascular diseases in the winter, a direct impact of periods with extreme temperature, and possibly mental and physiological effects (eg D‐vitamin) related to absence of daylight in winter, as well as social and psychological factors related to Christmas and New Year holidays^1^. However, influenza is recognised as one of the main contributing factors of winter excess mortality.

A common method to assess winter excess mortality estimates, based on predefined periods with no or ignorable influenza activity, is by using the Serfling method^2^, ^3^. The baseline for the Serfling model, which is usually fitted with a cyclic variation over the year, can be compared with the observed number of deaths, and the difference (the residual) presented as an estimate of excess mortality. Since 2009, the network for European monitoring of excess mortality for public health action (EuroMOMO) has monitored weekly excess all‐cause mortality^4^, using this method. Using this method, excess mortality may be an over‐ or underestimation of influenza‐associated mortality, depending on the impact of non‐influenza morbidity on mortality during the winter.

To obtain specific estimates of influenza‐associated mortality, parameters representing influenza activity can be included in a time series model, instead of excluding periods with influenza activity from the estimation of baseline mortality^3^, ^5^, ^6^, ^7^. In this approach, excess mortality associated with influenza is determined directly from influenza activity data and not as a residual, which will also be affected by non‐influenza factors.

Within the EuroMOMO network, we developed a model to estimate influenza‐associated mortality based on influenza activity in the population while controlling for extreme ambient temperatures, the FluMOMO model. This model can be used at the national level or at the European level to support risk assessment and health planning in connection with seasonal influenza epidemics and in a pandemic situation, and has the potential for timely, in‐season use.

The objective of this study was to describe the statistical model, FluMOMO, and apply it to estimate influenza‐associated mortality in Denmark for the seasons 2010/11 to 2016/17.

## 2 MATERIALS AND METHODS

First, we describe the FluMOMO model and consider how the influenza activity and extreme ambient temperatures variables were defined. Secondly, we describe data sources, and finally analytical methods.

### 2.1 The FluMOMO model

The model is a multivariable time series model with weekly number of deaths as dependent variable. We use a multiplicative Poisson regression with overdispersion and ISO‐week as time unit. Influenza activity (IA) and extreme temperature (ET) are included as independent variables. The residual variation is post‐regression corrected for skewness by applying a ⅔‐power correction^8^

A general formulation of the regression:

$$\begin{array}{cc} l(X_{t})= & \beta_{01}*{\text{baseline}}_{t}+\sum_{\mathrm{dIA}}\left(\sum_{s}\beta_{2\mathrm{dIA},s}*{\text{IA}}_{t-\mathrm{dIA},s}\right)\\ & +\sum_{\mathrm{dET}}\left(\sum_{p}\beta_{3\mathrm{dET},p}*{\text{ET}}_{t-\mathrm{dET},p}\right)+\varepsilon_{t} \end{array}$$

where ***l*** is the link function (ie logarithm in a multiplicative model), *X* is the observed number of deaths and *t* is calendar week. Season‐year (s) is defined as the period from week 27 to week 26 the following year, and p represents 4 parameters reflecting extreme temperature.

The baseline consists of a trend and seasonality expressed as 2 sine waves of 1 year and half year periods: sin(2π*t/(365.25/7)) + cos(2π*t/(365.25/7)), sin(2π*t/((365.25/2)/7)) + cos(2π*t/((365.25/2)/7)), respectively. The significance of the trend and the 2 sine waves is tested according to predefined levels, see below.

We estimate a separate effect of IA for each season and not for the entire period. The rationale for this relates to the fact that the type of circulating influenza virus and the match with the influenza vaccine will vary from one season to another.

Four parameters (ET_t,1_ to ET_t,4_) for ambient temperature were included, representing extreme cold in the winter (week 40 to week 20 the following year), where it may be associated with excess deaths, and extreme cold in the summer (week 21‐39), where it may have a benign effect. Conversely, periods with extreme heat may be associated with excess mortality in the summer but a benign effect in the winter.

We include a lag‐effect of the explanatory variables, that is, a carry‐over effect of IA or ET from earlier weeks. Length of the lags is predefined as external parameters, and dIA and dET represent the delay in the formula.

The model estimates the baseline as well as the effect of IA and ET simultaneously, controlled for one another, that is, the baseline correlates to IA and ET.

#### 2.1.1 Expected number of deaths

The expected mean number of deaths including both IA and ET as explanatory variables will be

$$\begin{array}{cc} E(X_{t})= & l^{-1}[\beta_{01}*{\text{baseline}}_{t}+\sum_{\mathrm{dIA}}(\sum_{s}\beta_{2,\mathrm{dIA},s}*{\text{IA}}_{t-\mathrm{dIA},s})\\ & +\sum_{\mathrm{dET}}(\sum_{p}\beta_{3,\mathrm{dET},p}*{\text{ET}}_{t-\mathrm{dET},p})]. \end{array}$$

The expected baseline will be

$$\begin{array}{cc} E({\text{baseline}}_{t})= & E\left(B_{t}\right)=l^{-1}[\beta_{01}*{\text{baseline}}_{t}+\sum_{\mathrm{dIA}}(\sum_{s}\beta_{2,\mathrm{dIA},s}*0)\\ & +\sum_{\mathrm{dET}}(\sum_{p}\beta_{3,\mathrm{dET},p}*0)]. \end{array}$$

Expected mean number of deaths if only IA or ET had an effect will be

$$\begin{array}{cc} E({\text{IA}}_{t})= & l^{-1}[\beta_{01}*{\text{baseline}}_{t}+\sum_{\mathrm{dIA}}(\sum_{s}\beta_{2,\mathrm{dIA},s}*{\text{IA}}_{t-\mathrm{dIA},s})\\ & +\sum_{\mathrm{dET}}(\sum_{p}\beta_{3,\mathrm{dET},p}*0)] \end{array}$$

$$\begin{array}{cc} E({\text{ET}}_{t})= & l^{-1}[\beta_{01}*{\text{baseline}}_{t}+\sum_{\mathrm{dIA}}(\sum_{s}\beta_{2,\mathrm{dIA},s}*0)\\ & +\sum_{\mathrm{dET}}(\sum_{p}\beta_{3,\mathrm{dET},p}*{\text{ET}}_{t-\mathrm{dET},p})]. \end{array}$$

**Figure 1:**
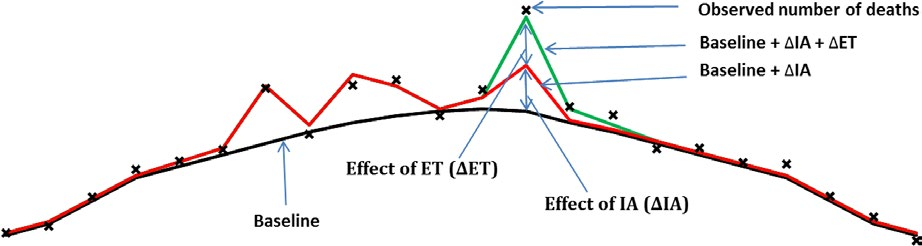
Baseline, effect of influenza activity and extreme temperature (ET)

Hence, the expected mean excess number of deaths associated with IA_t_ and ET_t_ will be (Figure 1):

$$E\left(\Delta {\text{IA}}_{t}\right)=E\left({\text{IA}}_{t}\right)-E\left(B_{t}\right)$$

$$E\left(\Delta {\text{ET}}_{t}\right)=E\left({\text{ET}}_{t}\right)-E\left(B_{t}\right).$$

For post‐estimation ⅔‐power correction of residual variation and cumulated estimates see Appendix A.

### 2.2 Influenza activity (IA)

A commonly used syndromic indicator of IA is the frequency of influenza‐like illness (ILI), measured for example, through a sentinel network of general practitioners (GP). ILI can be expressed as a percentage of consultations with ILI (consultation%). If the GPs’ catchment population is known, ILI can be expressed as an incidence. Should information on ILI be unavailable, acute respiratory illness (ARI) may be used as an alternative indicator.

In Denmark, ILI is reported as the number of ILI consultations by age group, but only the total number of consultations. Hence, we do not have a denominator for each age group's consultation%. However, assuming consultations have a constant distribution between age groups through a season, but vary between seasons, using the total number of consultations as the denominator in each age group, the consultation% in each age group will be proportional to the pattern of the correct consultation% over a specific season, thus providing valid estimates of the effect of IA, as the effect of IA is estimated separately for each season. Therefore, we used the total number of consultations as the denominator for all age groups.

Another indicator of IA is the percentage of influenza‐positive samples (positive%). In Denmark, all influenza samples are registered on a personal level, that is, with known age for each person, in a national centralised database^9^. For this study, we considered more than one sample from the same person, on the same date, as one sample. If at least one sample tested positive for influenza, we considered the sample as a positive sample independent of the test result of other samples taken that day. If a person had a positive sample, then all following samples in that season were excluded.

Influenza‐like illness may overestimate IA, as other respiratory infections may cause ILI. On the other hand, % positive may not fully reflect the intensity of IA in the population. Therefore, Goldstein et al^10^, ^11^ suggested using ILI × positive% (Goldstein index) as an indicator of IA.

We applied 3 indicators of IA in the FluMOMO model: ILI consultation%, positive% and the Goldstein index.

### 2.3 Extreme ambient temperatures

Danish weather stations register daily average temperature as well as daily minimum and maximum temperature. Averages over each of these daily temperatures, weighted by the populations in the 5 Danish regions, were used as the overall Danish daily temperature, and minimum and maximum temperature for that day. Weekly temperature as well as weekly minimum and maximum temperature was calculated as the weekly mean of each of the overall daily temperatures, respectively. Using each of these mean weekly temperatures, we estimated the expected weekly temperature, and expected minimum and maximum temperatures, in general linear models with a yearly seasonal variation, fitted as a sine wave.

**Figure 2:**
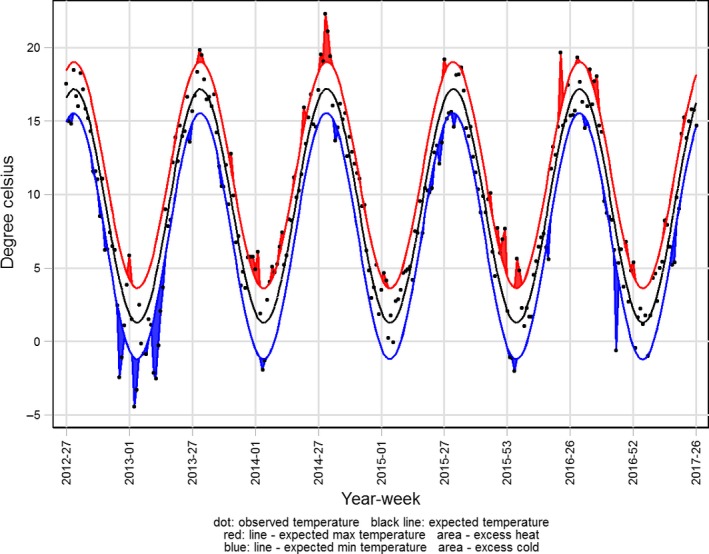
Weekly mean of average daily ambient temperatures in Denmark

Weeks with extreme temperatures were defined as weeks with a mean temperature above the expected maximum temperature or below the expected minimum temperature (Figure 2). Nominal values were defined as how far the observed temperature was below or above the expected minimum or maximum.

### 2.4 Data sources

#### 2.4.1 Deaths

Individual notifications of all‐cause dates of deaths and dates of birth were obtained from the Danish Civil Registry.

#### 2.4.2 Influenza activity

The Danish sentinel surveillance system for influenza is a voluntary system of GPs providing weekly reports (week 40‐20) on the total number of consultations and age‐specific numbers of ILI consultations to Statens Serum Institut. Any ILI consultations which took place outside of week 40‐20 were set to 0.

Electronic data on all patients swabbed at the general practitioners (GP) or at hospitals and tested for influenza virus by PCR were retrieved from the Danish Microbiology Database (MiBa)^12^, where these samples have been registered since 2010.

#### 2.4.3 Ambient temperatures

Data on daily temperatures registered at Danish weather stations was downloaded from the National Oceanic and Atmospheric Administration Online Climate Data Directory^13^.

#### 2.4.4 Population

The size of the Danish population by age at the start of every quarter of the year was downloaded from Statistics Denmark^14^. The weekly size of each age group specific population was achieved by linear interpolation.

### 2.5 Analyses

Analyses were performed separately for the age groups 0‐4, 5‐14, 15‐64 and 65+ years of age, as well as for all ages.

A fatal outcome after infection with influenza virus will normally occur after a time‐delay, where co‐morbidity and complications may contribute. On this basis, we applied a 2 weeks lag for both IA and ET.

We included a trend and a half‐yearly cycle if they contributed on a 5% level (*P* < .05), while yearly seasonality was included on a 10% level.

There may be other changes in mortality over calendar time than seasonality. Therefore, a trend is included in the baseline. Long‐term changes may be modelled using a non‐linear trend, for example, a spline. However, non‐linear trends tend to be less stable at the ends, and if we intend to use the model for monitoring, interest will be in the ends, especially the right end, which informs the most recent impact. As the model potentially can be used for in‐season and near timely monitoring, we have limited the model to use a linear trend.

When estimating over more than 5 seasons, the linear trend assumption did not always hold (likelihood‐ratio test between a spline and a linear trend), and including <3 seasons seems to make the fit less stable (most estimates become insignificant, because of low statistical power). Hence, we used 5 seasons’ estimation periods. Furthermore, when estimating results for a season, this must not depend on future observations, that is, following seasons, only on the present and preceding seasons. As we only have complete data from 2010 and onwards, using 5 seasons’ estimation periods, results for the seasons 2010/11 to 2013/14 can only be estimated using data from the preceding seasons and should therefore be interpreted with caution. We separated the analyses over the period 2010/11 to 2016/17 in 3 estimation periods. The first estimation period was from 2010/11 to 2014/15, reporting results for the 2014/15 season and also reporting results for the 2010/11 to 2013/14 seasons. The second estimation period was from 2011/12 to 2015/16 reporting results for the 2015/16 season, and finally the third estimation period was from 2012/13 to 2016/17, reporting the 2015/16 results.

Weeks with a negative IA‐effect were after estimation excluded from reported results, in line with a common practice in estimation of influenza‐associated mortality due to the challenge of interpretation of a non‐negative effect of influenza on mortality. However, unrestricted results are shown in Appendix B.

All analyses were performed using Stata 14.2 MP. The FluMOMO code in Stata and R is freely downloadable from the EuroMOMO website (http://www.EuroMOMO.eu).

## 3 RESULTS

**Figure 3:**
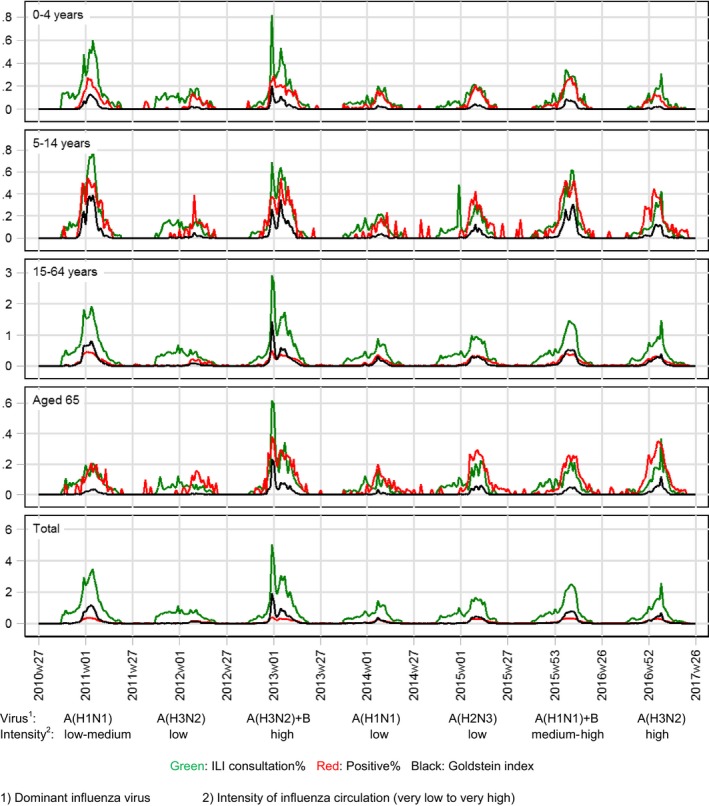
Weekly influenza activity indicators

During the seasons 2010/11 to 2016/17, around 146 (4.1%) of all Danish GPs participated in the sentinel surveillance, and a median of 15 822 (range 8129‐31 565) samples was tested for influenza virus per season. Figure 3 shows the 3 indicators of influenza activity (IA): ILI (consultation%), positive% and Goldstein index.

**Figure 4:**
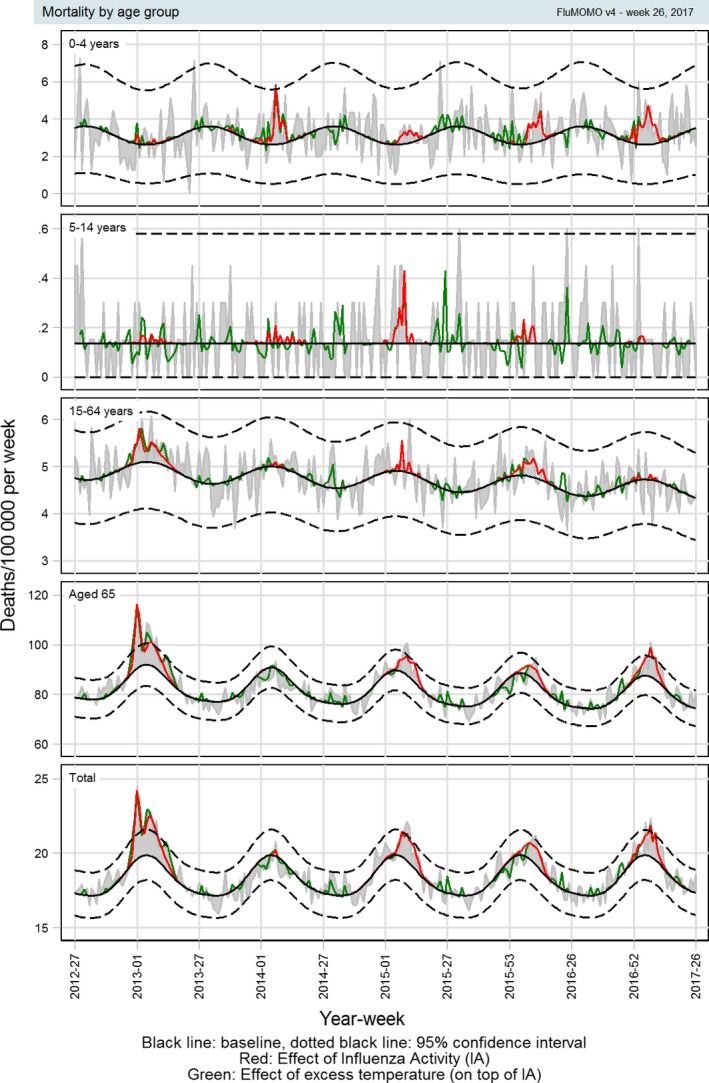
All‐cause mortality rates using the FluMOMO model with Goldstein index

**Figure B1:**
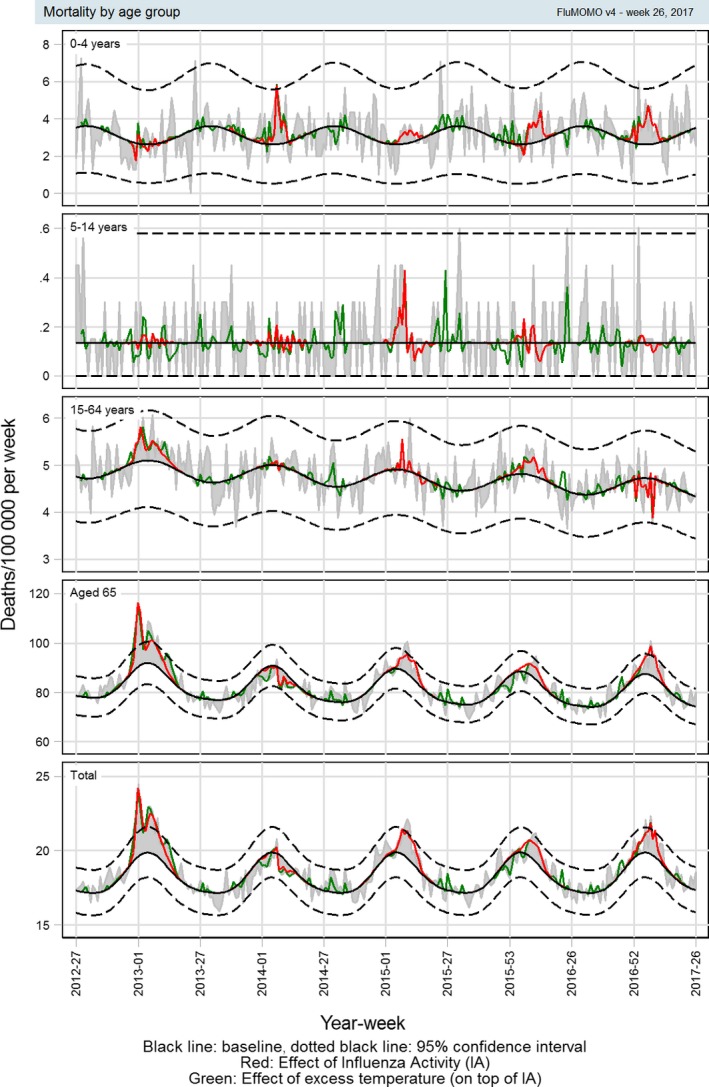
All‐cause mortality rates using the FluMOMO model with Goldstein index, where life‐saving effects of IA are included

After fitting the model, there were no indications of neither heteroscedasticity nor residual autocorrelation. The model had the same level of information criteria (AIC and BIC) across the different IA's, but varied between the age groups. AIC's for the 4 age groups (0‐4, 5‐14, 15‐64, ≥65) and for all ages were 5.49, 2.66, 8.33, 10.01 and 10.22, and for BIC −969.5, −1015.1, −983.2, −954.0 and −956.53. The fit of the model is illustrated in Appendix B (Figure B1). Figure 4 shows the outcome of the model, where effects of IA are restricted to be positive. The figure shows baseline, mortality rates associated with influenza using Goldstein index as IA indicator and mortality rates associated with ET over the 5 seasons 2012/13 to 2016/17 used to estimate the 2016/17 influenza‐associated mortality.

**Figure 5:**
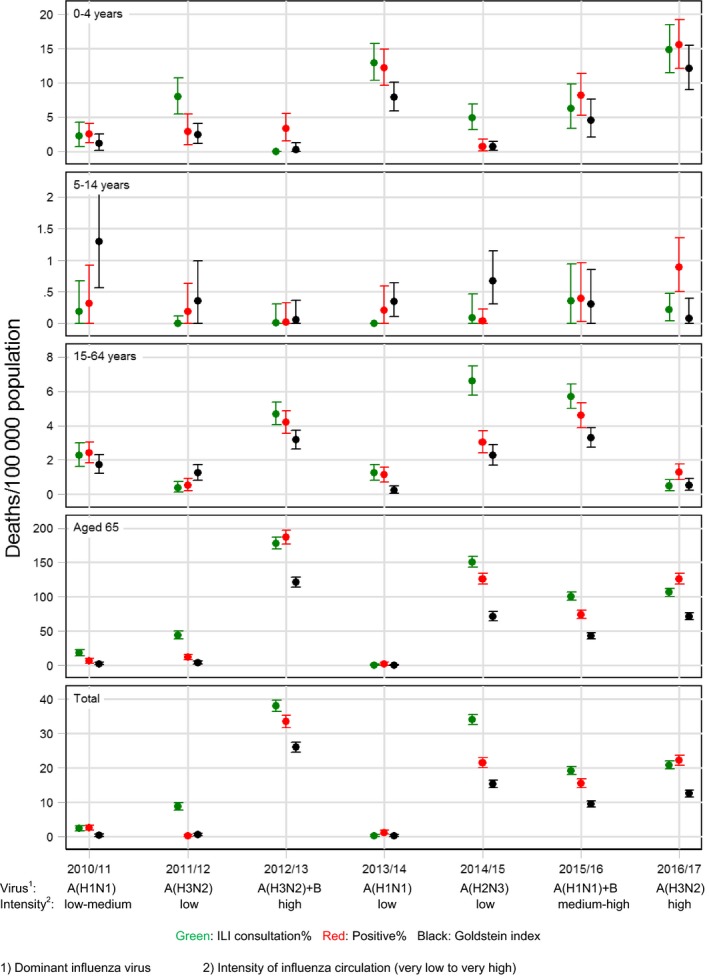
Influenza activity associated winter (week 40 to week 20 the following year) mortality rates using the FluMOMO model

Mortality rates varied depending on the IA indicator used, with a median mortality rate over the seasons 2010/11 to 2016/17 for all ages of 17.6 (range: 0.0‐36.2) deaths per 100 000 per year using ILI, 14.1 (range: 0.3‐31.6) using positive% and 8.3 (range: 0.0‐25.0) using Goldstein index. These figures correspond to a median of 1104 (range: 2‐2064), 805 (range: 14‐1771) and 474 (range: 0‐1399) deaths, respectively (Table 1). Number of deaths and mortality rates by age group for each of the different IA indicators are shown in Table 1 and illustrated in Figure 5.

**Table 1:** Influenza‐associated mortality rates based on different influenza activity indicators

Season^a^	Death	Mortality^b^	Death	Mortality^b^	Death	Mortality^b^	Death	Mortality^b^	Death	Mortality^b^
Age:	All ages		0‐4 years		5‐14 years		15‐64 years		Aged 65	
ILI (Consultation percentage)										
2010/11	118	2.12 (1.38‐2.96)	7	2.23 (0.47‐4.70)	1	0.19 (0.19‐0.19)	221	6.09 (5.09‐7.14)	166	17.52 (17.52‐17.52)
2011/12	494	8.85 (7.71‐10.04)	25	7.88 (4.95‐11.23)	0	‐	245	6.78 (5.86‐7.74)	436	44.38 (38‐36‐50.69)
2012/13	2064	36.82 (34.74‐38.93)	0	‐	0	‐	327	9.03 (7.91‐10.2)	1736	171.94 (161.36‐182.74)
2013/14	2	0.03 (0.03‐0.03)	38	12.69 (8.67‐17.18)	0	‐	206	5.67 (4.74‐6.65)	2	0.15 (0.15‐0.15)
2014/15	1861	32.80 (31.04‐34.59)	14	4.60 (2.30‐7.38)	1	0.09 (0.09‐0.09)	406	11.12 (10.08‐12.19)	1545	145.78 (137.12‐154.61)
2015/16	1004	17.56 (16.06‐19.10)	16	5.32 (2.51‐8.76)	2	0.33 (0.33‐0.33)	193	5.26 4.34‐6.23)	983	90.74 (83.19‐98.51)
2016/17	1231	21.38 (19.86‐22.96)	20	6.56 (3.56‐10‐13)	1	0.22 (0.22‐0.22)	20	0.54 (0.54‐0.54)	1152	104.49 (96.39‐112.38)
Median	1004	17.56	16	5.32	1	0.09	221	6.09	983	90.74
Positive percentage										
2010/11	133	2.38 (1.58‐3.29)	8	2.53 (2.53‐2.53)	13	1.93 (1.00‐3.04)	88	2.43 (1.70‐3.26)	55	5.81 (2.73‐9.58)
2011/12	14	0.25 (0.25‐0.25)	9	2.82 (1.03‐5‐14)	3	0.46 (0.00‐1.24)	20	0.56 (0.56‐0.56)	116	11.77 (8.28‐15.65)
2012/13	1771	31.58 (29.61‐33.58)	10	3.22 (1.16‐5.88)	1	0.10 (0.00‐0.54)	151	4.17 (3.34‐5.07)	1811	179.39 (168.89‐190.14)
2013/14	21	0.37 (0.37‐0.37)	37	12.28 (12.28‐12.28)	3	0.50 (0.05‐1.19)	44	1.21 (1.21‐1.21)	3	0.27 (0.27‐0.27)
2014/15	1090	19.22 (17.76‐20.72)	2	0.55 (0.55‐0.55)	4	0.66 (0.66‐0.66)	105	2.87 (2.21‐3.58)	1264	119.22 (11.39‐127.22)
2015/16	805	14.07 (12.69‐15.50)	23	7.64 (7.64‐7.64)	2	0.37 (0.01‐0.99)	154	4.20 (3.38‐5.08)	719	66.37 (59.71‐73.26)
2016/17	1310	22.77 (21.22‐24.34)	48	15.91 (11.62‐20.63)	6	0.87 (0.87‐0.87)	51	1.38 (0.86‐1.98)	1347	122.15 (114.26‐130.22)
Median	805	14.07	10	3.22	3	0.50	88	2.43	719	66.37
Goldstein index										
2010/11	19	0.34 (0.34‐0.34)	4	1.12 (0.00‐3.21)	9	1.29 (0.49‐2.32)	65	1.80 (1.80‐1.80)	21	2.26 (0.30‐5.18)
2011/12	39	0.70 (0.26‐1.28)	8	2.44 (0.64‐4.88)	2	0.35 (0.00‐1.17)	47	1.30 (0.77‐1.92)	42	4.28 (1.77‐7.42)
2012/13	1399	24.95 (23.12‐26.83)	1	0.29 (0.00‐1.90)	0	0.07 (0.00‐0.53)	115	3.17 (2.38‐4.03)	1176	116.46 (107.08‐126.10)
2013/14	0	0.01 (0.01‐0.01)	24	7.92 (7.92‐7.92)	2	0.34 (0.00‐1.05)	9	0.25 (0.25‐0.25)	1	0.07 (0.07‐0.07)
2014/15	822	14.49 (13.13‐15.89)	2	0.52 (0.00‐2.24)	5	0.68 (0.68‐0.68)	84	2.29 (1.66‐2.99)	724	68.26 (61.52‐75.23)
2015/16	474	8.29 (7.14‐9.50)	12	4.21 (1.66‐7.45)	2	0.29 (0.29‐0.29)	107	2.92 (2.19‐3.72)	416	38.39 (32.7‐44.38)
2016/17	751	13.04 (11.75‐14.38)	36	12.14 (8.18‐16.60)	1	0.08 (0.08‐0.08)	17	0.46 (0.46‐0.46)	767	69.54 (62.77‐76.53)
Median	474	8.29	8	2.44	2	0.34	65	1.8	416	38.39

a Season: week 40 to week 20 the following year.

b Deaths per 100 000 per year (95% confidence interval).

## 4 DISCUSSION

Infection with the influenza virus may often lead to exacerbation of underlying chronic conditions or to secondary bacterial infections^15^. Therefore, influenza is often not recorded as the primary cause of death, and using cause‐specific data as an outcome may lead to underestimation of influenza‐associated mortality. Furthermore, in most countries, cause of death is available with large delays. Therefore, timely estimation of influenza‐associated mortality should be based on all‐cause mortality, although this outcome may be associated with loss of some specificity. The present work started as an initiative within the EuroMOMO network, with the aim of obtaining a direct and timely estimation of mortality associated with influenza.

In the present study based on Danish data, the model generally captures the background seasonal mortality pattern well. However, for some weeks, the estimated effect of IA was negative. For small, insignificant negative effects, this will be within the statistical variation. A decision to exclude these weeks will cause a slight overestimation of influenza‐associated mortality. However, negative effects that are statistically significant or seen over a long period may indicate a problem with a poor fit of the model, for example if the baseline is too high. Presently, there is no proper solution to this problem, which warrants more attention. From a biological standpoint, a negative effect of influenza can be interpreted as a “protective effect,” which is implausible. To overcome this, many modellers present negative values as a nought^3^, ^16^. Along these lines, we excluded weeks with negative values from influenza‐associated mortality. This choice is convenient because it facilitates dissemination of a public health message of influenza‐associated mortality, but it warrants a more critical review. The effect of excluding weeks with negative effects of IA is shown in appendix B, showing results without this restriction.

Other respiratory pathogens are not directly included in the model, even though they may affect winter mortality. If they should be included in the model, care has to be taken to avoid collinearity, in particular when lags are included, as a lagged effect of one pathogen may be confounded by a direct impact of another pathogen^5^, ^15^. Additionally, if included in the model, potential interactions may complicate the model and blur the estimates. However, this should not prevent further investigation.

Another factor of importance in the seasonal variation in mortality is ambient temperature or more precisely deviation from expected temperature, for example, cold snaps in the winter or heat waves in the summer^17^, ^18^. We have controlled influenza‐associated mortality for temperature, but the pattern of the effect of temperature on mortality has to be investigated further to be able to report valid estimates of this effect.

We estimated Danish influenza‐associated mortality using 3 indicators of influenza activity (Table 1 and Figure 5). All 3 indicators show a similar pattern. ILI possibly overestimates influenza mortality as ILI may be caused by other respiratory pathogens. Using positive% as indicator for IA will reduce the effect of other respiratory pathogens, but may not, as ILI, reflect the dynamic in the population. A combination of the 2, the Goldstein index, will reflect both the dynamic of transmission and limit overestimation due to other pathogens^10^, ^11^. Mortality associated with influenza based on the Goldstein index seems to be the most appropriate for Danish data, primarily because it is conservative. Using the Goldstein index, we found a median influenza‐associated mortality rate of 8.2 per 100 000 population which is substantially lower—and probably more realistic—than a previous applied methodology that obtained a median mortality of 26 per 100 000 in the period 1994‐2010^19^. Though, the introduction of free vaccinations for elderly and risk groups may have contributed to this decline. However, this should be evaluated further including data from other EuroMOMO countries.

The Danish influenza data have 2 limitations. It is voluntary for GPs to participate in the reporting of ILI, and the distribution of GP's participating is not random. Secondly, sampling of influenza testing may be biased towards more samples taken at hospitals, and therefore may overestimate the population positive%. These limitations in ILI and positive% will introduce some uncertainty in the Goldstein index. However, we observed no bias in the estimated effects that may have been due to this.

The influenza season 2010/11, the first after the 2009/10 pandemic, was dominated by influenza A(N1H1)pdm09 with low to medium intensity^20^, and very low influenza‐associated mortality, except for children 5‐14 year of age, corroborating the observation from the early waves of the pandemic^21^. The season 2011/12 was exceptional with a very low influenza A(H3N2) activity and low mortality for all ages, even though there was significant activity in parts of Europe including Norway and Sweden^22^. In the season 2012/13, Denmark experienced a long period with high influenza activity dominated by A(H3N2), but also B by the end of the season^23^. The outcome was a marked excess influenza mortality among the elderly. Influenza activity and mortality were low in the A(H1N1) dominated 2013/14 season, although raised among children below 5 years of age^24^. In the following season, 2014/15, A(H3N2) influenza started to circulate late with low intensity. However, many patients received intensive care, and mortality was high among the elderly^25^, ^26^. In the rather long 2015/16 season, influenza first came as A(N1H1)pdm09 and was followed by B. Intensity was relative high and many patients received intensive care^27^. Mortality was moderately raised in all ages, higher than the previous A(H1N1) seasons, also among the elderly. The later could be due to influenza B. The 2016/17 A(H3N2) dominated season started early giving a long season, with a high intensity. The number of influenza‐related admissions was the highest since 2010/11, but with relatively few patients receiving intensive care^28^. Mortality was high among the elderly.

We present a time series model, the FluMOMO model, with the aim of estimating mortality associated with influenza, and thereby contributing to the need for severity assessment of influenza. We used all‐cause mortality as the outcome, because all‐cause mortality data are readily available in most countries and have previously been found to be applicable for assessing the total impact of influenza on mortality^15^. The model fitted well, but was subject to some limitations, including the handling of negative estimates, that should be further investigated. The parametrisation of the effect of extreme temperatures should be further developed to provide robust estimates of excess mortality associated with extreme temperatures.

The 3 indicators of IA, ILI (consultation%), positive% and Goldstein index, showed similar patterns. We recommend the Goldstein index, because it provided conservative estimates, and is a more valid representation of influenza activity than indicators from syndromic surveillance (ILI/ARI) or laboratory indicators that do not encompass information about circulation in the community.

We have shown than FluMOMO has the potential to provide robust and standardised seasonal estimates of mortality associated with influenza. Furthermore, if data are available, the model may also provide in‐season estimates. One of the challenges with timely estimates is the delay in registration. The EuroMOMO algorithm includes a compensation for delay in registration of deaths. Hence, countries participating in the EuroMOMO network can use these delay‐adjusted numbers of deaths. This implies that the FluMOMO model potentially can provide timely estimates of influenza‐associated mortality, depending on timeliness of the influenza indicators (ILI and positive%).

In conclusion, the FluMOMO can be used to model the impact of influenza on mortality in almost real time, and we encourage to include the FluMOMO model as a tool in “the toolbox of influenza surveillance,” in particular to support severity assessment. Meanwhile, the model, including the use of different IA indicators, should be further evaluated.

## CONFLICT OF INTERESTS

The authors declare that they have no competing interests.

## AUTHORS’ CONTRIBUTION

JN formulated the FluMOMO model, did the analyses and was the lead writer. TGV and KM wrote part of background and discussion, and contributed with ideas and comments to methods and results. All authors approved the whole manuscript.

## Figures and Tables

**Figure B2:**
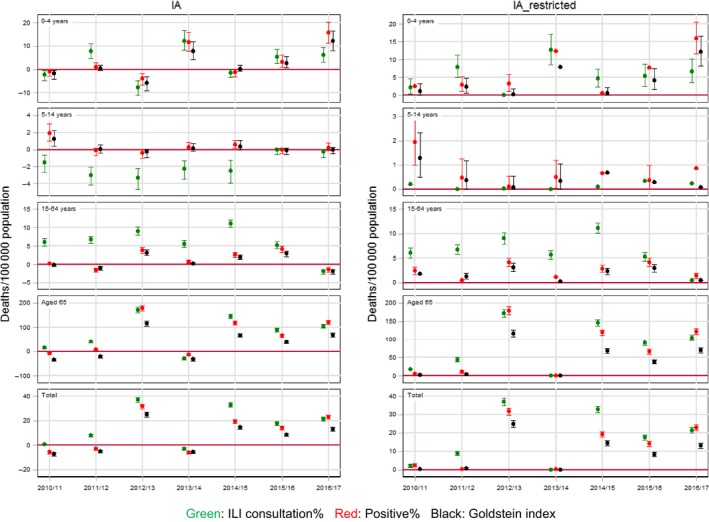
Influenza activity associated winter (week 40 to week 20 the following year) mortality rates using the FluMOMO model

## APPENDIX A

### POST‐ESTIMATION ⅔‐POWER CORRECTION OF RESIDUAL VARIANCE

Formulation of the ⅔‐power variance corrected residual variance (RVar), that is, the uncertainty or confidence intervals of the estimated mean effects:

Using the delta‐method, the residual ⅔‐power variances of ∆IA_t_ or ∆ET_t_ becomes

$$\begin{array}{cc} \text{RVar}(\Delta {\text{IA}}_{t}) & =\text{Var}\left(E{\left({\text{IA}}_{t}\right)}^{\frac{2}{3}}-E{\left(B_{t}\right)}^{\frac{2}{3}}\right)=\text{Var}\left(E{\left({\text{IA}}_{t}\right)}^{\frac{2}{3}}\right)+\text{Var}\left(E{\left(B_{t}\right)}^{\frac{2}{3}}\right)\\ & \approx {\left(\frac{2}{3}*E{\left({\text{IA}}_{t}\right)}^{\frac{2}{3}-1}\right)}^{2}*\text{Var}\left(E({\text{IA}}_{t})\right)+{\left(\frac{2}{3}*E{\left(B_{t}\right)}^{\frac{2}{3}-1}\right)}^{2}*\text{Var}\left(E(B_{t})\right) \end{array}$$

where *E*(*B*_t_) is the estimated baseline number of deaths and *E*(IA_t_) is the expected number of deaths had the effect of ET_t_ been zero. Var(E(B_t_)) and Var(E(IA_t_)) are the regression variances. However, in a regression with a link function ***l,*** these are “only” known at the ***l***‐scale. Using the link function log, as we do, the regression variance become

$$\text{Var}\left(E\left(X_{t}\right)\right)=\left(\text{exp}({\text{Var}}_{\log}\left(E\left(X_{t}\right)\right))-1\right)*\text{exp}\left(2\text{log}\left(E\left(X_{t}\right)\right)+{\text{Var}}_{\log}\left(E\left(X_{t}\right)\right)\right),$$

where Var_log_(*E*(*X*_t_)) is the regression variance on the log‐scale. Hence

$$\begin{array}{cc} \text{RVar}(\Delta {\text{IA}}_{t}) & \approx (\frac{2}{3}*E{\left({\text{IA}}_{t}\right)}^{\frac{2}{3}-1}{)}^{2}*(\text{exp}\left({\text{Var}}_{\log}\left(E\left({\text{IA}}_{t}\right)\right)\right)-1)*\text{exp}(2*\text{log}\left(E\left({\text{IA}}_{t}\right)\right)\\ & +{\text{Var}}_{\log}\left(E\left({\text{IA}}_{t}\right)\right))+(\frac{2}{3}*E{\left(B_{t}\right)}^{\frac{2}{3}-1}{)}^{2}*(\text{exp}\left({\text{Var}}_{\log}\left(E\left(B_{t}\right)\right)\right)-1)\\ & *\text{exp}(2*\text{log}\left(E\left(B_{t}\right)\right)+{\text{Var}}_{\log}\left(E\left(B_{t}\right)\right)) \end{array}$$

and likewise for ET_t_:

$$\begin{array}{cc} \text{RVar}(\Delta {\text{ET}}_{t}) & \approx (\frac{2}{3}*E{\left({\text{ET}}_{t}\right)}^{\frac{2}{3}-1}{)}^{2}*(\text{exp}\left({\text{Var}}_{\log}\left(E\left({\text{ET}}_{t}\right)\right)\right)-1)\text{exp}(2*\text{log}\left(E\left({\text{ET}}_{t}\right)\right)\\ & +{\text{Var}}_{\log}\left(E\left({\text{ET}}_{t}\right)\right))+(\frac{2}{3}*E{\left(B_{t}\right)}^{\frac{2}{3}-1}{)}^{2}*(\text{exp}\left({\text{Var}}_{\log}\left(E\left(B_{t}\right)\right)\right)-1)\\ & \text{exp}(2*\text{log}\left(E\left(B_{t}\right)\right)+{\text{Var}}_{\log}\left(E\left(B_{t}\right)\right)) \end{array}$$

Confidence intervals are then calculated as:

$${\text{CI}}_{\left(1-\alpha \right)}\left(\Delta X_{t}\right)={\left(\Delta X_{t}^{\frac{2}{3}}\pm z_{\left(1-\alpha \right)}\text{RVAR}\left(\Delta X_{t}\right)\right)}^{\frac{3}{2}}$$

where *X*_t_ may be IA or ET, respectively.

### CUMULATED NUMBER OF EXCESS DEATHS

The mean cumulated excess number of deaths over s weeks will be

$$\sum_{s}E\left(\Delta {\text{IA}}_{t}\right)=\sum_{s}E\left({\text{IA}}_{t}\right)-\sum_{s}E\left(B_{t}\right)$$

$$\sum_{s}E\left(\Delta {\text{ET}}_{t}\right)=\sum_{s}E\left({\text{ET}}_{t}\right)-\sum_{s}E\left(B_{t}\right).$$

Assuming independence between weeks, that is, no residual autocorrelation, then the ⅔‐power variance corrected residual variance of the cumulated excess deaths over s weeks will be

$$\begin{array}{cc} \text{RVar}(\sum_{s}\Delta {\text{IA}}_{t}) & =\text{Var}((\sum_{s}E\left({\text{IA}}_{t}\right){)}^{\frac{2}{3}}-(\sum_{s}E\left(B_{t}\right){)}^{\frac{2}{3}})=\text{Var}((\sum_{s}E\left({\text{IA}}_{t}\right){)}^{\frac{2}{3}})\\ & +\text{Var}((\sum_{s}E\left(B_{t}\right){)}^{\frac{2}{3}})\\ & \approx (\frac{2}{3}*(\sum_{s}E\left({\text{IA}}_{t}\right){)}^{\frac{2}{3}-1}{)}^{2}*\text{Var}(\sum_{s}E\left({\text{IA}}_{t}\right))\\ & +(\frac{2}{3}*(\sum_{s}E\left(B_{t}\right){)}^{\frac{2}{3}-1}{)}^{2}*\text{Var}(\sum_{s}E\left(B_{t}\right))\\ & =(\frac{2}{3}*(\sum_{s}E\left({\text{IA}}_{t}\right){)}^{\frac{2}{3}-1}{)}^{2}*\sum_{s}\text{Var}(E\left({\text{IA}}_{t}\right))\\ & +(\frac{2}{3}*(\sum_{s}E\left(B_{t}\right){)}^{\frac{2}{3}}-1{)}^{2}*\sum_{s}\text{Var}(E\left(B_{t}\right))\\ & =(\frac{2}{3}*(\sum_{s}E\left({\text{IA}}_{t}\right){)}^{\frac{2}{3}-1}{)}^{2}*\sum_{s}(\text{exp}\left({\text{Var}}_{\log}\left(E\left({\text{IA}}_{t}\right)\right)\right)-1)\\ & *\text{exp}(2*\text{log}\left(E\left({\text{IA}}_{t}\right)\right)+{\text{Var}}_{\log}\left(E\left({\text{IA}}_{t}\right)\right))\\ & +(\frac{2}{3}*{\left(\sum_{s}E\left(B_{t}\right)\right)}^{\frac{2}{3}-1}{)}^{2}*\sum_{s}(\text{exp}\left({\text{Var}}_{\log}\left(E\left(B_{t}\right)\right)\right)-1)\\ & *\text{exp}(2*\text{log}\left(E\left(B_{t}\right)\right)+{\text{Var}}_{\log}\left(E\left(B_{t}\right)\right)) \end{array}$$

$$\begin{array}{cc} \text{RVar}(\sum_{s}\Delta {\text{ET}}_{t}) & \approx (\frac{2}{3}*{\left(\sum_{s}E\left({\text{ET}}_{t}\right)\right)}^{\frac{2}{3}-1}{)}^{2}*\sum_{s}(\text{exp}\left({\text{Var}}_{\log}\left(E\left({\text{ET}}_{t}\right)\right)\right)-1)\\ & *\text{exp}(2*\text{log}\left(E\left({\text{ET}}_{t}\right)\right)+{\text{Var}}_{\log}\left(E\left(\text{ET}{+}_{t}\right)\right))\\ & +(\frac{2}{3}*{\left(\sum_{s}E\left(B_{t}\right)\right)}^{\frac{2}{3}-1}{)}^{2}*\sum_{s}(\text{exp}\left({\text{Var}}_{\log}\left(E\left(B_{t}\right)\right)\right)-1)\\ & *\text{exp}(2*\text{log}\left(E\left(B_{t}\right)\right)+{\text{Var}}_{\log}\left(E\left(B_{t}\right)\right)) \end{array}.$$

Confidence intervals are then calculated as:

$${\text{CI}}_{\left(1-\alpha \right)}\left(\sum_{s}\Delta X_{t}\right)={\left((\sum_{s}{\text{EX}}_{t}{)}^{\frac{2}{3}}\pm z_{\left(1-\alpha /2\right)}\text{RVAR}\left(\sum_{s}\Delta X_{t}\right)\right)}^{\frac{3}{2}}-\left(\sum_{s}{\text{EB}}_{t}\right)$$

where EB is baseline and *X*_t_ may be IA or ET, respectively.

## APPENDIX B

### B.1

**Table B1:** Influenza‐associated mortality rates based on different influenza activity (IA) indicators, where life‐saving effects of IA have not been excluded

Season^a^	Death	Mortality^b^	Death	Mortality^b^	Death	Mortality^b^	Death	Mortality^b^	Death	Mortality^b^
Age:	All ages		0‐4 years		5‐14 years		15‐64 years		Aged 65	
ILI (Consultation percentage)										
2010/11	33	0.60 (0.16 to 1.19)	−7	−2.22 (−4.69 to −0.47)	−10	−1.53 (−2.65 to −0.64)	219	6.05 (5.05 to 7.10)	149	15.72 (11.26 to 20.65)
2011/12	445	7.97 (6.87 to 9.12)	25	7.75 (4.84 to 11.09)	−20	−3.04 (−4.15 to −2.04)	242	6.68 (5.76 to 7.63)	409	41.66 (35.77 to 47.84)
2012/13	2064	36.82 (34.74 to 38.93)	−24	−7.60 (−11.03 to −4.61)	−22	−3.32 (−4.62 to −2.18)	327	9.03 (7.91 to 10.20)	1736	171.94 (161.36 to 182.74)
2013/14	−166	−2.94 (−3.84 to −2.12)	37	12.26 (8.30 to 16.71)	−15	−2.27 (−3.44 to −1.27)	205	5.63 (4.70 to 6.61)	−302	−29.11 (−34.87 to −23.71)
2014/15	1861	32.80 (31.04 to 34.59)	−4	−1.38 (−3.40 to −0.10)	−16	−2.47 (−3.92 to −1.25)	406	11.11 (10.07 to 12.18)	1545	145.78 (137.12 to 154.61)
2015/16	1004	17.55 (16.05 to 19.09)	16	5.32 (2.51 to 8.75)	0	−0.06 (−0.53 to 0.21)	193	5.26 (4.24 to 6.23)	983	90.74 (83.19 to 98.51)
2016/17	1231	21.38 (19.86 to 22.94)	18	6.07 (3.16 to 9.56)	−2	−0.25 (−0.94 to 0.06)	−70	−1.89 (−2.57 to −1.29)	1152	104.49 (96.79 to 112.38)
Median	1004	17.55	16	5.32	−15	2.27	219	6.05	983	90.74
Positive percentage										
2010/11	−323	−5.81 (−7.00 to −4.69)	−3	−0.93 (−2.89 to 0.04)	13	1.92 (0.99 to 3.03)	6	0.18 (−0.02 to 0.59)	−67	−7.07 (−11.06 to −373)
2011/12	−169	−3.03 (−3.91 to −2.23)	4	1.12 (0.03 to 2.95)	−1	−0.11 (−0.70 to 0.20)	−55	−1.52 (−2.13 to −0.98)	71	7.27 (4.67 to 10.63)
2012/13	1767	31.51 (29.54 to 33.51)	−12	−3.81 (−6.60 to −1.59)	−3	−0.43 (−1.02 to −0.05)	138	3.80 (2.99 to 4.67)	1811	179.39 (168.86 to 190.14)
2013/14	−332	−5.89 (−6.99 to −4.86)	35	11.55 (7.72 to 15.86)	2	0.24 (−0.04 to 0.83)	28	0.78 (0.35 to 1.30)	−129	−12.44 (−16.70 to −8.63)
2014/15	1086	19.14 (17.68 to 20.63)	−3	−1.17 (−3.05 to −0.04)	4	0.56 (0.16 to 1.10)	94	2.58 (1.94 to 3.27)	1263	119.16 (11.33 to 127.16)
2015/16	805	14.07 (12.69 to 15.50)	9	3.19 (0.95 to 6.19)	0	−0.04 (−0.46 to 0.21)	153	4.17 (3.35 to 5.04)	718	66.31 (59.65 to 73.19)
2016/17	1308	22.72 (21.18 to 24.30)	47	15.75 (11.48 to 20.45)	1	0.19 (−0.07 to 0.76)	−53	−1.42 (−2.03 to −0.89)	1337	121.25 (113.38 to 129.30)
Median	805	14.07	4	1.12	1	0.19	28	0.78	718	66.31
Goldstein index										
2010/11	−406	−7.30 (−854 to −6.13)	−6	−1.80 (−4.15 to −0.23)	8	1.24 (0.45 to 2.25)	−5	−0.13 (−0.53 to 0.05)	−324	−34.12 (−40.49 to −28.12)
2011/12	−276	−4.94 (−5.96 to −3.99)	1	0.35 (−0.43 to 1.96)	0	0.02 (−0.38 to 0.57)	−36	−0.98 (−1.55 to −0.51)	−204	−20.79 (−25.81 to −16.15)
2012/13	1399	25.95 (23.12 to 26.83)	−18	−5.85 (−9.06 to −3.15)	−2	−0.28 (−0.89 to 0.01)	115	3.17 (2.38 to 4.03)	1176	116.46 (107.08 to 126.10)
2013/14	−308	−5.46 (−6.55 to −4.43)	23	7.75 (4.21 to 11.94)	1	0.13 (−0.15 to 0.72)	5	0.15 (−0.03 to 0.52)	−339	−32.72 (−38.61 to −21.16)
2014/15	822	14.49 (13.13 to 15.89)	1	0.23 (−0.56 to 1.73)	2	0.38 (0.00 to 1.07)	72	1.98 (1.38 to 2.64)	724	68.26 (61.52 to 75.23)
2015/16	474	8.29 (7.14 to 9.50)	8	2.70 (0.62 to 5.58)	0	−0.07 (−0.57 to 0.20)	106	2.89 (2.16 to 2.68)	416	38.39 (32.70 to 44.38)
2016/17	751	13.04 (11.75 to 14.38)	36	12.06 (8.10 to 16.50)	0	−0.03 (−0.49 to 0.28)	−72	−1.95 (−2.63 to −1.35)	767	68.54 (62.77 to 76.53)
Median	474	8.29	1	0.35	0	0.02	5	0.15	416	38.39

a Season: week 40 to week 20 the following year.

b Deaths per 100 000 per year (95% confidence interval).
